# Cationic oxides and dioxides of modified sugarcane bagasse beads with applications as low-cost sorbents for direct red 28 dye

**DOI:** 10.1038/s41598-024-51934-7

**Published:** 2024-01-13

**Authors:** Pornsawai Praipipat, Pimploy Ngamsurach, Nantikorn Libsittikul, Chawanluk Kaewpetch, Punpruksa Butdeesak, Wachira Nachaiperm

**Affiliations:** 1 Department of Environmental Science, Faculty of Science, Khon Kaen University, Khon Kaen, 40002 Thailand; 2 Environmental Applications of Recycled and Natural Materials (EARN) Laboratory, Khon Kaen University, Khon Kaen, 40002 Thailand

**Keywords:** Engineering, Materials science

## Abstract

The direct red 28 (DR28) dye contamination in wastewater blocks the transmission of light into the water body resulting in the inability to photosynthesize by aquatic life. In addition, it is difficult to break down and persist in the environment, and it is also harmful to aquatic life and water quality because of its aromatic structure. Thus, wastewater contaminated with dyes is required to treat before releasing into the water body. Sugarcane bagasse beads (SBB), sugarcane bagasse modified with titanium dioxide beads (SBBT), sugarcane bagasse modified with magnesium oxide beads (SBBM), sugarcane bagasse modified with aluminum oxide beads (SBBA), and sugarcane bagasse modified with zinc oxide beads (SBBZ) for DR28 dye removal in aqueous solution, and they were characterized with several techniques of BET, FESEM-FIB, EDX, FT-IR, and the point of zero charges (pH_pzc_). Their DR28 dye removal efficiencies were examined through batch tests, adsorption isotherms, and kinetics. SBBM had the highest specific surface area and pore volume, whereas its pore size was the smallest among other materials. The surfaces of SBB, SBBM, SBBT, and SBBA were scaly sheet surfaces with an irregular shape, whereas SBBZ was a coarse surface. Oxygen, carbon, calcium, chloride, sodium, O–H, C–H, C=O, C=C, and C–O–C were found in all materials. The pH_pzc_ of SBB, SBBT, SBBM, SBBA, and SBBZ were 6.57, 7.31, 10.11, 7.25, and 7.77. All materials could adsorb DR28 dye at 50 mg/L by more than 81%, and SBBM had the highest DR28 dye removal efficiency of 94.27%. Langmuir model was an appropriate model for SBB, whereas Freundlich model was a suitable model for other materials. A pseudo-second-order kinetic model well described their adsorption mechanisms. Their adsorptions of the DR28 dye were endothermic and spontaneous. Therefore, they were potential materials for adsorbing DR28 dye, especially SBBM.

## Introduction

Dye-contaminated wastewater affects to be toxic to aquatic organisms because it has an aromatic structure difficult to degrade, and the colored particles may block the transmission of light into the water body. As a result, aquatic plants and algae are unable to photosynthesize. Furthermore, the lack of oxygen in water sources affects life in water and destroys the scenery which is offensive to the onlookers^1^. Many industries of dye, pigment, paint, paper, printing, cosmetics, and textile widely use dyes in their product manufacturing, especially direct dyes are popularly used for long-lasting cellulose and lignin dyeing^2^. Direct red 28 (DR28) dye is also popularly used for dyeing cotton in many industries, so wastewater with contaminated DR28 dyes is recommended to be treated before discharging for environmental safety.

The treatment methods of dyes are coagulation-flocculation, chemical oxidation, electrochemistry, ion exchange, ozonation, photochemistry, adsorption, and biological process^3^. However, adsorption is a favored method for adsorbing dyes because it is the effective method, easy operation, suitable cost, and offering several adsorbents^4^. In addition, the main criteria of good adsorbents are required as environmentally friendly adsorbent, easy access, cheap cost, and cost-effective use, so the agricultural waste is one option that corresponds to these requirements above. Many agricultural wastes have been used for removing several dyes shown in Table 1. Many studies reported in Table 1 have applied the sugarcane bagasse to eliminate dyes of reactive blue 19, methyl red, and basic red 2, reactive blue 4^5–8^, so they can affirm the sugarcane bagasse's ability to adsorb several dyes. However, the development of sugarcane bagasse to deal with the specific pollutant targets with the high concentration strength of industrial wastewater also needs more investigation.

**Table 1:** The agricultural wastes with or without modifications for removing various dyes**.**

Materials	Dyes	Dose (g)	Time (min)	Temp (°C )	pH	Conc. (mg/L)	Volume (mL)	*q*_m_ (mg/g)	Refs
Non-modification									
White dragon fruit peel	Direct red 28	0.02	180	25	5.02	0–1,000	10	59.30	^10^
Potato peel	Direct red 80	2	60	30	2	20–200	100	27.78	^11^
Potato peel	Methylene blue	2	10	30	7	20–200	100	97.08	^11^
Groundnut shell (activated carbon)	Direct red 1	0.1	20	28	2	10–50	100	5.11	^12^
Almond shell	Crystal violet	0.2	90	20	6	20–200	40	12.20	^13^
Corn silk	Reactive blue 19	0.25	1440	25	2	10–500	50	60.60	^14^
Corn silk	Reactive red 218	0.25	1440	25	2	10–500	50	51.60	^14^
Rice husk	Direct red 28	0.5	10	30	4	20–100	15	1.58	^15^
Sugarcane bagasse (biochar)	Reactive blue 19	0.2	360	25	2	50–1000	50	58.10	^5^
Sugarcane bagasse	Methyl red	0.4	180	26	6	50–200	100	5.66	^6^
Sugarcane bagasse	Basic red 2	1	1020	25	10	5–40	250	58.85	^7^
Bagasse (beads)	Reactive blue 4	2	720	70	3	30–90	100	3.17	^8^
Modification									
Sugarcane bagasse treated by phosphoric acid (H_3_PO_4_)	Methyl red	0.4	180	26	6	50–250	100	10.98	^6^
Sugarcane bagasse treated by sulfuric acid (H_2_SO_4_)	Basic red 2	1	1020	25	10	5–40	250	54.82	^7^
Sugarcane bagasse treated by NaOH	Basic red 2	1	1020	25	10	5–40	250	62.88	^7^
Sugarcane bagasse MgO/N-doped active carbon	Methyl orange	0.05	100	30	–	130–170	150	384.61	^16^
Sugarcane bagasse modified with iron oxide (Fe_3_O_4_)	Methylene Blue	16.5	360	25	8.4	1–10	25	12.28	^17^
Bagasse beads with mixed iron (III) oxide-hydroxide	Reactive blue 4	3	540	70	3	30–90	100	3.77	^8^
Bagasse beads with mixed zinc oxide	Reactive blue 4	3	720	60	3	30–90	100	3.18	^8^
Bagasse beads mixed titanium dioxide	Reactive blue 4	1.5	900	30	3	30–90	100	6.04	^9^
Bagasse beads mixed magnesium oxide	Reactive blue 4	2.5	900	30	3	30–90	100	5.55	^9^
Bagasse beads mixed aluminum oxide	Reactive blue 4	3	900	30	3	30–90	100	3.41	^9^

Many methods of acid treatment, alkaline treatment, and metal oxide modifications are used to increase the abilities of sugarcane bagasse materials for dye removals also illustrated in Table 1. In previous studies, sugarcane bagasse beads modified with titanium dioxide (TiO_2_), magnesium oxide (MgO), aluminum oxide (Al_2_O_3_), and zinc oxide (ZnO) have been used for removing RB4 dye^8,9^; however, no one used them to remove DR28 dye. As a result, their comparison results need to confirm the abilities of sugarcane bagasse beads modified with those metal oxides for removing several anionic dyes. Therefore, this current study attempts to investigate the abilities of sugarcane bagasse beads with or without metal oxide modifications for removing DR28 dye to understand how the addition of metal oxide with different types affects DR28 dye, and which one offers the highest DR28 dye removal.

In this study, sugarcane bagasse beads (SBB), sugarcane bagasse beads modified with titanium dioxide (SBBT), sugarcane bagasse beads modified with magnesium oxide (SBBM), sugarcane bagasse beads modified with aluminum oxide (SBBA), and sugarcane bagasse beads modified with zinc oxide (SBBZ) were synthesized for investigating their characterizations and DR28 dye removal efficiencies. Brunauer–Emmett–Teller (BET), Field emission scanning electron microscopy and focus ion beam (FESEM-FIB), Energy dispersive X-ray spectrometer (EDX), and Fourier transform infrared spectroscopy (FT-IR) were used for identifying their specific surface area, pore volumes, pore sizes, surface structures, chemical elements, and chemical functional groups. In addition, their points of zero charge (pH_pzc_) were also investigated to recognize their surface charges. The affecting factors of dosage, contact time, temperature, pH, and concentration were examined by batch tests, and their adsorption isotherms and kinetics were also determined by nonlinear models of Langmuir, Freundlich, Temkin, Dubinin–Radushkevich, pseudo-first-order kinetic, pseudo-second-order kinetic, Elovich, and intra-particle diffusion for describing their adsorption patterns and mechanisms. The thermodynamic study was also investigated to understand the temperature effect on their DR28 dye removals.

## Material and method

### Raw material and preparation

Sugarcane bagasse was taken from the local market in Khon Kaen province, Thailand. Before use, it was washed with tap water to remove contaminations, and then it was dried in a hot air oven (Binder, FED 53, Germany) at 80 °C for 24 h. Then, it was ground, sieved in size of 125 µm, and kept in a desiccator called sugarcane bagasse powder (SBP)^8^.

### Chemicals

All chemicals used in this study were analytical grades (AR) without purification. They were titanium dioxide (TiO_2_) (Loba, India), magnesium oxide (MgO) (RCI Labscan, Thailand), aluminum oxide (Al_2_O_3_) (Kemaus, New Zealand), zinc oxide (ZnO) (QRëC, New Zealand), sodium alginate (NaC_6_H_7_O_6_) (Merck, Germany), calcium chloride dihydrate (CaCl_2_·2H_2_O) (RCI Labscan, Thailand), direct red 28 (DR28) dye (C_32_H_22_N_6_Na_2_O_6_S_2_) (Sigma-Aldrich, Germany), 0.1 M HCl (RCI Labscan, Thailand), and 0.1 M NaOH (RCI Labscan, Thailand). The pH adjustments used 0.5% nitric acid (HNO_3_) (Merck, Germany) and 0.5% NaOH (RCI Labscan, Thailand).

### Dye solution preparation

The dye solutions are prepared from the stock solution of direct red 28 (DR28) dye of 100 mg/L concentration.

### Material synthesis

**Figure 1:**
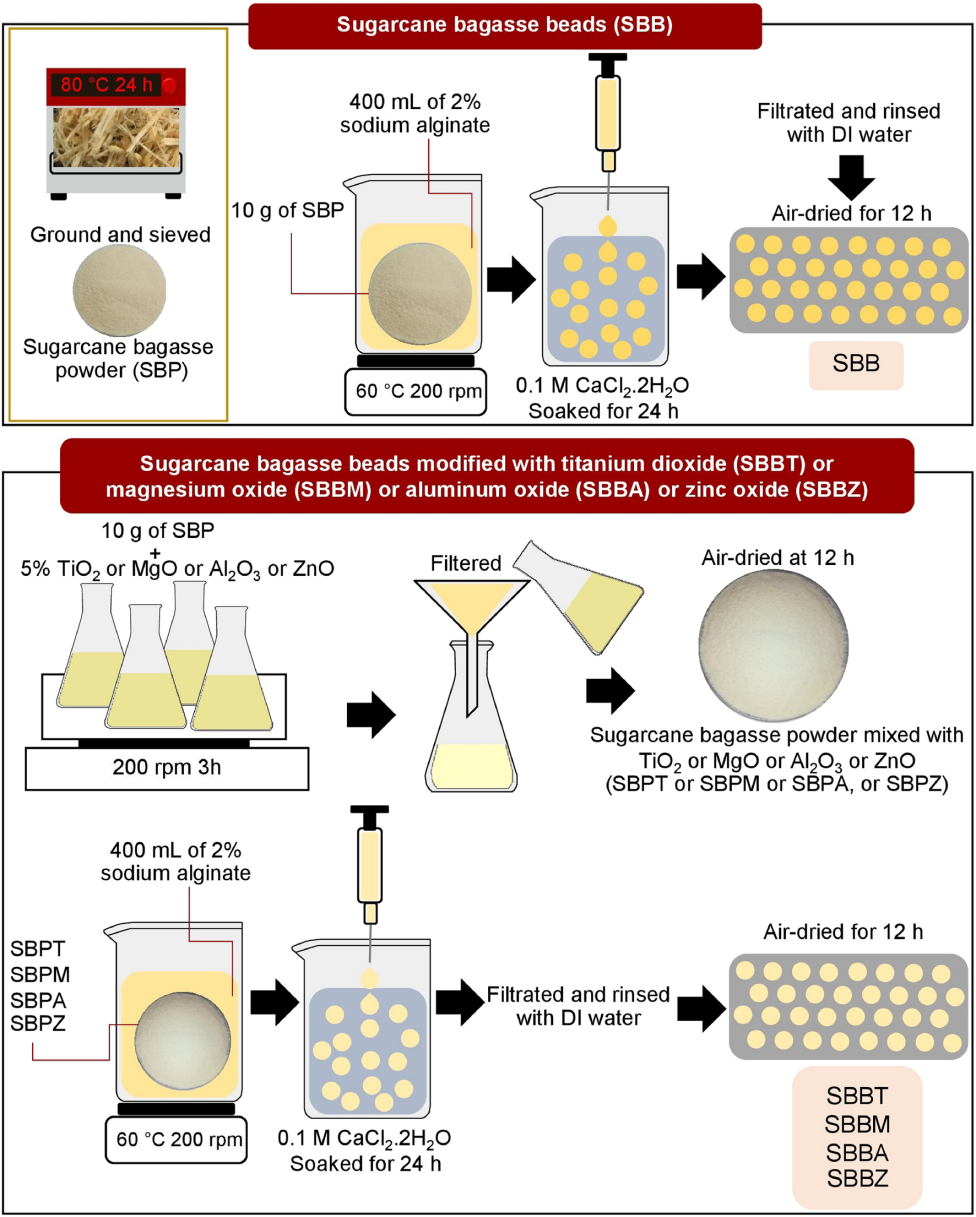
The synthesis of SBB, SBBT, SBBM, SBBA, and SBBZ.

The material synthesis methods are mentioned from the study of Ngamsurach et al.^8^, Praipipat et al.^9^, and Praipipat et al.^18^, and the flow diagrams are illustrated in Fig. 1. The details are described below:

#### The synthesis of sugarcane bagasse beads (SBB)

Firstly, 10 g of SBP were added to a 1000 mL beaker containing 400 mL of 2% NaC_6_H_7_O_6_, then they were heated by a hot plate (Ingenieurbüro CAT, M. Zipperer GmbH, M 6, Germany) at 60 °C with a stable stirring speed of 200 rpm until homogeneous mixed. Next, they were contained into a syringe with a needle (1.2 mm × 25 mm), and they were dropwise into 250 mL of 0.1 M CaCl_2_·2H_2_O and soaked for 24 h for a bead setting. Then, they were filtrated, rinsed with DI water, and air-dried at room temperature for 12 h. Finally, they were kept in a desiccator before use called sugarcane bagasse beads (SBB).

#### The synthesis of sugarcane bagasse beads modified with titanium dioxide (SBBT) or magnesium oxide (SBBM) or aluminum oxide (SBBA) or zinc oxide (SBBZ)

Firstly, 10 g of SBP were added to a 250 mL Erlenmeyer flask containing 160 mL of 5% (w/v) TiO_2_ or MgO or Al_2_O_3_ or ZnO solution prepared by the deionized water, and they were homogeneously mixed by an orbital shaker (GFL, 3020, Germany) of 200 rpm for 3 h. Next, they were filtered, air-dried at room temperature for 12 h, and kept in a desiccator called sugarcane bagasse powder mixed with TiO_2_ or MgO or Al_2_O_3_ or ZnO (SBPT or SBPM or SBPA, or SBPZ). Then, SBPT or SBPM or SBPA, or SBPZ were added to a 1000 mL beaker containing 400 mL of 2% NaC_6_H_7_O_6_, then they were heated by a hot plate at 60 °C with a stable stirring speed of 200 rpm until homogeneous mixed. Next, they were contained into a syringe with a needle (1.2 mm × 25 mm), and they were dropwise into 250 mL of 0.1 M CaCl_2_·2H_2_O and soaked for 24 h for a bead setting. Then, they were filtrated, rinsed with DI water, and air-dried at room temperature for 12 h. Finally, they were kept in a desiccator before use called sugarcane bagasse modified with titanium dioxide beads (SBBT), sugarcane bagasse modified with magnesium oxide beads (SBBM), sugarcane bagasse modified with aluminum oxide beads (SBBA), and sugarcane bagasse modified with zinc oxide beads (SBBZ).

### Material characterizations

The material characterizations on the specific surface area, pore volumes, pore sizes, surface structures, chemical elements, and chemical functional groups of SBB, SBBT, SBBM, SBBA, and SBBZ were investigated by Brunauer–Emmett–Teller (BET), Field emission scanning electron microscopy and focus ion beam (FESEM-FIB) with Energy dispersive X-ray spectrometer (EDX) (FEI, Helios NanoLab G3 CX, USA), and Fourier transform infrared spectroscopy (FT-IR) (Bruker, TENSOR27, Hong Kong).

### *The point of zero charge (pH*_*pzc*_*)*

The method of the points of zero charge of SBB, SBBT, SBBM, SBBA, and SBBZ for DR28 dye adsorptions is mentioned from the studies of Praipipat et al.^18,19^ which was the pH drift method by preparing 0.1 M NaCl solutions with pH values from 2 to 12 by using 0.1 M HCl and 0.1 M NaOH. Then, 2 g/L of SBB or SBBT or SBBM or SBBA, or SBBZ were added to 50 mL of 0.1 M NaCl solution contained in 250 mL Erlenmeyer flask, and it was shaken at 150 rpm for 24 h at room temperature by an orbital shaker. Finally, the final pH of the sample was measured by a pH meter (Mettler Toledo, SevenGo with InLab 413/IP67, Switzerland) and calculated ∆pH (pH_final_–pH_initial_) to determine the point of zero charge (pH_pzc_).

### Batch experiments

The affecting factors of dose (5–30 g/L), contact time (3–18 h), temperature (20–50 °C), pH (3–11), and concentration (30–90 mg/L) with the control condition of initial DR28 dye concentration of 50 mg/L, a sample volume of 100 mL, and a shaking speed of 150 rpm by using an incubator shaker (New Brunswick, Innova 42, USA)^8,9,20^ on DR28 dye removal efficiencies of SBB, SBBT, SBBM, SBBA, and SBBZ were investigated through a series of batch experiments which referred from the previous study of Praipipat et al.^18^ Their optimum conditions were chosen from the lowest dose or contact time or temperature or pH or concentration with obtaining the highest DR28 dye removal efficiencies^9^. UV–VIS Spectrophotometer (UH5300, Hitachi, Japan) with a wavelength of 497 nm was used for analyzing dye concentrations, and the triplicate experiments were investigated to verify the results and report the average value. Dye removal efficiency in the percentage and dye adsorption capacity is calculated following Eqs. (1)–(2):

1 $${\text{Dye removal efficiency }}\left( \% \right) \, = \, \left( {\left( {C_{0} - C_{{\text{e}}} } \right)/C_{0} } \right) \, \times \, 100$$

2 $${\text{Dye adsorption capacity }}\left( {q_{e} } \right) \, = (C_{0} - C_{{\text{e}}} )V/m$$

where *C*_e_ is the dye concentration at equilibrium (mg/L), *C*_0_ is the initial dye concentration (mg/L)*, q*_e_ is the capacity of dye adsorption on adsorbent material at equilibrium (mg/g)*, V* is the sample volume (L), and *m* is the amount of adsorbent material (g).

### Adsorption isotherms

The adsorption patterns of SBB, SBBT, SBBM, SBBA, and SBBZ were determined by using nonlinear Langmuir, Freundlich, Temkin, and Dubinin–Radushkevich models. Langmuir model is monolayer adsorption, and Freundlich model represents multilayer adsorption^21,22^. Temkin model refers to the heat of adsorption with decreasing from the increase of coverage adsorbent, and Dubinin–Radushkevich model is used to determine the adsorption mechanism between physisorption and chemisorption^23,24^. Their adsorption isotherms were calculated by Eqs. (3)–(6)^20–24^:

Langmuir isotherm:

3 $$q_{e} = \, q_{{\text{m}}} K_{L} C_{{\text{e}}} /1 + K_{{\text{L}}} C_{{\text{e}}}$$

Freundlich isotherm:

4 $$q_{{\text{e}}} = \, K_{{\text{F}}} C_{{\text{e}}}^{1/n}$$

Temkin isotherm:

5 $$q_{{\text{e}}} = RT/b_{{\text{T}}} {\text{ln}}A_{{\text{T}}} C_{{\text{e}}}$$

Dubinin–Radushkevich isotherm:

6 $$q_{e} = q_{{\text{m}}} {\text{exp}}( - K_{{{\text{DR}}}} \varepsilon^{2} )$$

where *q*_e_ is the capacity of dye adsorption on adsorbent material at equilibrium (mg/g), *q*_m_ is the maximum capacity of dye adsorption on adsorbent material (mg/g),* C*_e_ is the equilibrium of dye concentration (mg/L), *K*_L_ is Langmuir adsorption constant (L/mg), *K*_F_ is Freundlich constant of adsorption capacity (mg/g)(L/mg)^1/n^, and *n* is the constant depicting of the adsorption intensity. *R* is the universal gas constant (8.314 J/mol K), *T* is the absolute temperature (K), *b*_T_ is the constant related to the heat of adsorption (J/mol), *A*_T_ is the equilibrium binding constant corresponding to maximum binding energy (L/mg), *K*_DR_ is the activity coefficient related to mean adsorption energy (mol^2^/J^2^), and *ε* is the Polanyi potential (J/mol). Their graphs are plotted by *q*_e_ versus *C*_e_.

For adsorption isotherm experiments, 25 g/L and 18 h of SBB, or 15 g/L and 18 h of SBBT or 20 g/L and 6 h of SBBM, or 15 g/L and 12 h of SBBA, or 25 g/L and 12 h of SBBZ have added to 250 mL Erlenmeyer flasks with variable DR28 dye concentrations from 30 to 90 mg/L. The control condition of SBB or SBBT or SBBM or SBBA or SBBZ was a sample volume of 100 mL, a shaking speed of 150 rpm, pH 3, and a temperature of 35 °C.

### Adsorption kinetics

The adsorption rate and mechanism of SBB, SBBT, SBBM, SBBA, and SBBZ were determined by using nonlinear pseudo-first-order kinetic, pseudo-second-order kinetic, Elovich, and intra-particle diffusion models. The pseudo-first-order and pseudo-second-order kinetic models are the physisorption and chemisorption processes^25,26^. Elovich model is the chemical adsorption process with a heterogeneous surface, and the intra-particle diffusion model refers to the rate limiting in the adsorption process^27,28^. Their adsorption kinetics were calculated by Eqs. (7)–(10)^25–28^:

Pseudo-first-order kinetic model:

7 $$q_{{\text{t}}} = q_{{\text{e}}} (1 - e^{{ - k^{{k_{1} t}} }} )$$

Pseudo-second-order kinetic model:

8 $$q_{{\text{t}}} = \, k_{2} q_{{\text{e}}}^{2} t/\left( {1 + \, q_{{\text{e}}} k_{2} t} \right)$$

Elovich model:

9 $$q_{t} = \beta \;{\text{ln}}\;t + \beta \;{\text{ln}}\;\alpha$$

Intra-particle diffusion model:

10 $$q_{{\text{t}}} = k_{{\text{i}}} t^{0.5} + C_{{\text{i}}}$$

where *q*_e_ is the capacity of dye adsorption on adsorbent material at equilibrium (mg/g)*, q*_t_ is the capacity of dye adsorption on adsorbent material at the time (*t*) (mg/g), *k*_1_ is a pseudo-first-order rate constant (min^−1^), and *k*_2_ is a pseudo-second-order rate constant (g/mg min). *α* is the initial adsorption rate (mg/g min) and *β* is the extent of surface coverage (g/mg). *k*_i_ is the intra-particle diffusion rate constant (mg/g min^0.5^) and *C*_i_ is the constant that gives an idea about the thickness of the boundary layer (mg/g)^19,29^. Their graphs are plotted by *q*_t_ versus *t*.

For the kinetic experiments, 25 g/L of SBB or 15 g/L of SBBT or 20 g/L of SBBM or 15 g/L of SBBA, or 25 g/L of SBBZ were added to a 1000 mL beaker. The control condition of SBB or SBBT or SBBM or SBBA, or SBBZ was a sample volume of 1000 mL, DR28 dye concentrations of 50 mg/L, a shaking speed of 150 rpm, pH 3, and a contact time of 24 h^18^.

### Thermodynamic study

The temperature effect on DR28 dye adsorption capacities of SBB, SBBT, SBBM, SBBA, and SBBZ were investigated through thermodynamic studies in a range of 293.15–323.15 K, and their results were explained by three thermodynamic parameters of Gibb free energy (∆*G*°), standard enthalpy change (∆*H*°), and standard entropy change (∆*S*°). Equations (11)–(13) were used to calculate their parameters^18^.

11 $$\Delta G^\circ \, = \, - RT\;{\text{ln}}\;K_{{\text{c}}}$$

12 $${\text{ln}}\;K_{{\text{c}}} = \, - \Delta H^\circ /RT + \, \Delta S^\circ /R$$

13 $$\Delta G^\circ \, = \, \Delta H^\circ \, - T\Delta S^\circ$$

where *R* is the universal gas constant (8.314 J/mol K), *T* is the absolute temperature (K), and *K*_c_ is the equilibrium constant (L/mg). The values of ∆*H*° and ∆*S*° were calculated from the slope and intercept of the linear graph between ln *K*_c_ (*K*_c_ = *q*_e_/*C*_e_) and 1/*T*, and ∆*G*° is calculated from Eq. (13).

For the thermodynamic experiments, 25 g/L and 18 h of SBB, or 15 g/L and 18 h of SBBT or 20 g/L and 6 h of SBBM, or 15 g/L and 12 h of SBBA, or 25 g/L and 12 h of SBBZ were applied with temperatures of 293.15–323.15 K with the control condition of DR28 dye concentration of 50 mg/L, a sample volume of 100 mL, pH 3, and a shaking speed of 150 rpm^20^.

## Result and discussion

### BET

The specific surface area, pore volumes, and pore sizes of SBB, SBBT, SBBM, SBBA, and SBBZ are illustrated in Table 2. Their specific surface area and pore volume could be arranged from high to low of SBBM > SBBT > SBBA > SBBZ > SBB, and SBBM demonstrated the highest surface area and pore volume among other materials. Since magnesium oxide (MgO), titanium dioxide (TiO_2_), aluminum oxide (Al_2_O_3_), and zinc oxide (ZnO) have a high specific surface area by themselves, the specific surface area of prepared materials by those metal oxides have higher specific surface area than raw material. Moreover, the previous studies reported the specific surface area of MgO, TiO_2_, Al_2_O_3_, and ZnO were 60, 50, 40, and 30 m^2^/g, and they could be arranged in order from high to low of MgO > TiO_2_ > Al_2_O_3_ > ZnO^30,31^. As a result, it could support why SBBM had a higher surface area than other materials. Therefore, metal oxides of TiO_2_, MgO, Al_2_O_3_, and ZnO increased the specific area and pore volumes of materials from the formations of those metal oxides with sugarcane bagasse supported more active sites for capturing DR28 dye adsorptions similar reported by previous studies used the same metal oxides^9,18,20^. Moreover, other metal oxides of zinc oxide, iron(III) oxide-hydroxide, and goethite have also been used in previous studies supported this study that the raw materials with adding metal oxides increased the surface area and pore volume^18,32–36^. Since their pore sizes were more than 2 nm, they were classified as mesoporous materials by the International Union of Pure and Applied Chemistry (IUPAC) classification^37^.

**Table 2:** The specific surface area, pore volumes, and pore sizes of SBB, SBBT, SBBM, SBBA, and SBBZ.

Materials	Specific surface area (m^2^/g)* ± SD	Pore volume (cm^3^/g)** ± SD	Pore size (nm)** ± SD
SBB	14.734 ± 0.011	0.039 ± 0.002	6.495 ± 0.014
SBBT	51.376 ± 0.014	0.063 ± 0.002	3.685 ± 0.015
SBBM	57.241 ± 0.012	0.071 ± 0.001	3.364 ± 0.017
SBBA	43.485 ± 0.010	0.051 ± 0.003	4.052 ± 0.013
SBBZ	39.197 ± 0.016	0.045 ± 0.002	4.319 ± 0.012

*BET specific surface area.

**Barrett–Joyner–Halenda (BJH) method.

### FESEM-FIB and EDX

**Figure 2:**
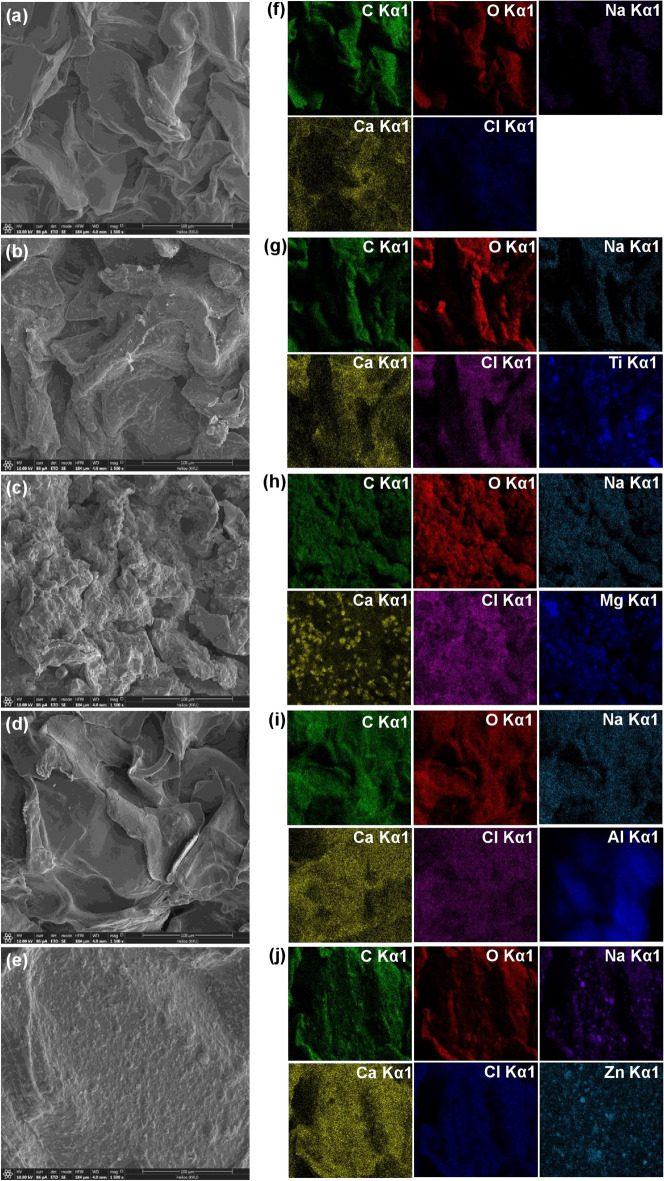
The surface morphologies and the chemical distributions by EDX mapping of (**a**, **f**) SBB, (**b**, **g**) SBBT, (**c**, **h**) SBBM, (**d**, **i**) SBBA, and (**e**, **j**) SBBZ.

For FESEM-FIB analysis, the surface morphologies at 1,500X magnification with 100 µm of SBB, SBBT, SBBM, SBBA, and SBBZ are demonstrated in Fig. 2a–e. The surfaces of SBB, SBBM, SBBT, and SBBA were scaly sheet surfaces and structures with an irregular shape similar to other studies reported^8,9^, whereas SBBZ had a coarse surface similar found in a previous study^8^.

For EDX analysis, the chemical elements of SBB, SBBT, SBBM, SBBA, and SBBZ are illustrated in Table 3, and their EDX mapping distributions are also demonstrated in Fig. 2f–j. Five main chemical elements of oxygen (O), carbon (C), calcium (Ca), chloride (Cl), and sodium (Na) were observed in all materials, whereas titanium (Ti), magnesium (Mg), aluminum (Al), and zinc (Zn) only detected in SBBT, SBBM, SBBA, and SBBZ, respectively because of addition of those metal oxides. In addition, the observations of Na, Ca, and Ca in all materials might be from the chemicals of sodium alginate and calcium chloride used in bead formations.

**Table 3:** The chemical elements of SBB, SBBT, SBBM, SBBA, and SBBZ.

Materials	Chemical elements (%wt) ± SD								
	O	C	Ca	Cl	Na	Ti	Mg	Al	Zn
SBB	45.6 ± 0.2	41.3 ± 0.1	8.1 ± 0.1	4.4 ± 0.1	0.6 ± 0.1	–	–	–	–
SBBT	42.7 ± 0.3	28.8 ± 0.2	6.5 ± 0.2	2.4 ± 0.2	0.9 ± 0.1	18.7 ± 0.2	–	–	–
SBBM	41.6 ± 0.1	33.3 ± 0.1	7.4 ± 0.3	3.5 ± 0.1	0.7 ± 0.1	–	13.5 ± 0.2	–	–
SBBA	35.4 ± 0.2	35.2 ± 0.2	6.7 ± 0.4	2.6 ± 0.1	0.8 ± 0.1	–	–	19.3 ± 0.2	–
SBBZ	32.2 ± 0.3	30.5 ± 0.2	7.9 ± 0.3	3.2 ± 0.2	0.9 ± 0.2	–	–	–	25.3 ± 0.3

### FT-IR

**Figure 3:**
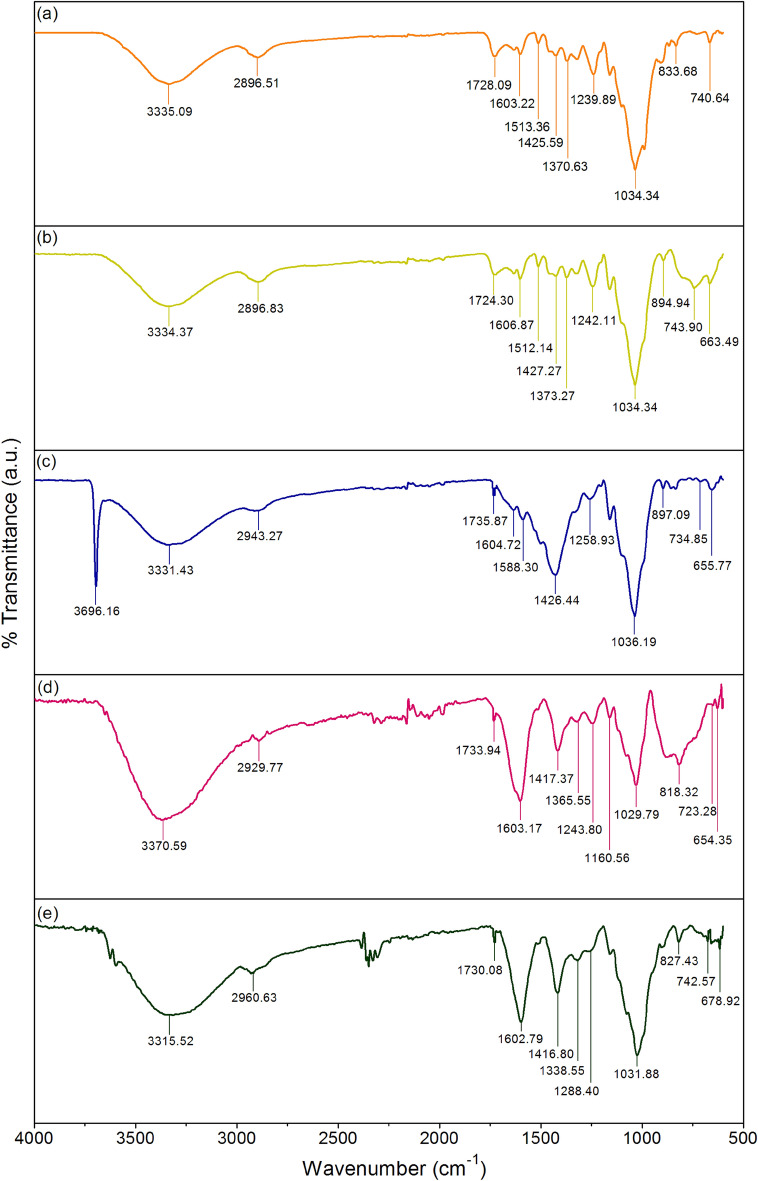
FT-IR spectra of (**a**) SBB, (**b**) SBBT, (**c**) SBBM, (**d**) SBBA, and (**e**) SBBZ.

The chemical functional groups of SBB, SBBT, SBBM, SBBA, and SBBZ are illustrated in Fig. 3a–e which they observed five main chemical functional groups of O–H, C–H, C=O, C=C, and C–O–C similar found in previous studies^8,9,29^. For O–H, it was the stretching water molecule, hydroxide groups of alcohol, phenol, and carboxylic acids^9^, and they were found in a range of 3310–3700 cm^−1^. For C–H, it referred to the bending of alkane (CH_2_), alkene (CH_3_), and aliphatic and aromatic groups of cellulose^38^ observed in a range of 2896–2960 cm^−1^. In addition, C–H also represented the stretching of CH_3_ in a range of 1330–1430 cm^−1^, and C–H was the bending of lignin and aromatic ring^39^ in a range of 720–750 cm^−1^. For C=O, it was the stretching of the carbonyl group, aldehyde, and ketone^39^ illustrated in a range of 1720–1740 cm^−1^. For C=C, it was the stretching of the aromatic ring in the lignin structure and the stretching of hemicellulose and cellulose^29^ which were found in ranges of 1500–1610 cm^−1^ and 810–900 cm^−1^, respectively. For C–O–C, it referred to the stretching of hemicellulose, cellulose, and sodium alginate^8^ in a range of 1020–1090 cm^−1^. Moreover, the functional groups of Ti–O–Ti, Mg–O, Al–O, and Zn–O were observed in SBBT, SBBM, SBBA, and SBBZ from the addition of titanium dioxide, magnesium oxide, aluminum oxide, and zinc oxide^18^ which were found at 663.49, 655.77, 654.35, and 678.92 cm^−1^, respectively.

### ***The point of zero charge (pH***_***pzc***_***)***

**Figure 4:**
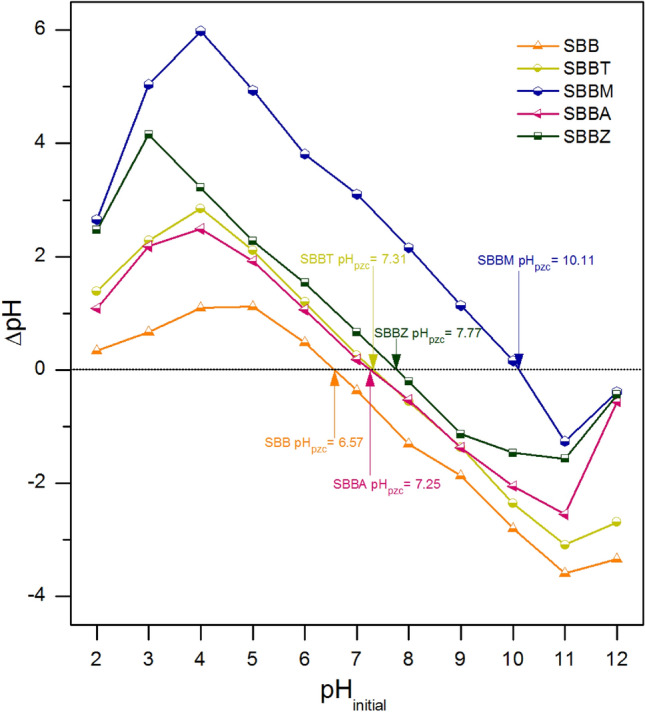
The points of zero charge of SBB, SBBT, SBBM, SBBA, and SBBZ.

The surface charges of SBB, SBBT, SBBM, SBBA, and SBBZ were determined by the point of zero charge (pH_pzc_) to expect which pH is preferred for DR28 dye adsorption of each material. Figure 4 is illustrated the pH_pzc_ of SBB, SBBT, SBBM, SBBA, and SBBZ which were 6.57, 7.31, 10.11, 7.25, and 7.77, and SBBM illustrated the highest pH_pzc_ among other materials similar found in a previous study^18^. Since the anionic dye should be adsorbed at a pH of solution (pH_solution_) less than pH_pzc_ because of the positively charged material surface, it can catch up DR28 dye molecule. On the other hand, DR28 dye adsorption is not favored at a pH_solution_ higher than pH_pzc_ because of the negatively charged material surface and the repulsion of the DR28 dye molecule. Therefore, DR28 dye adsorptions of each material should take place at a pH of solution less than its pH_pzc_ (pH_solution_ < pH_pzc_)^18,40^.

### Batch experiments

#### The effect of dosage

**Figure 5:**
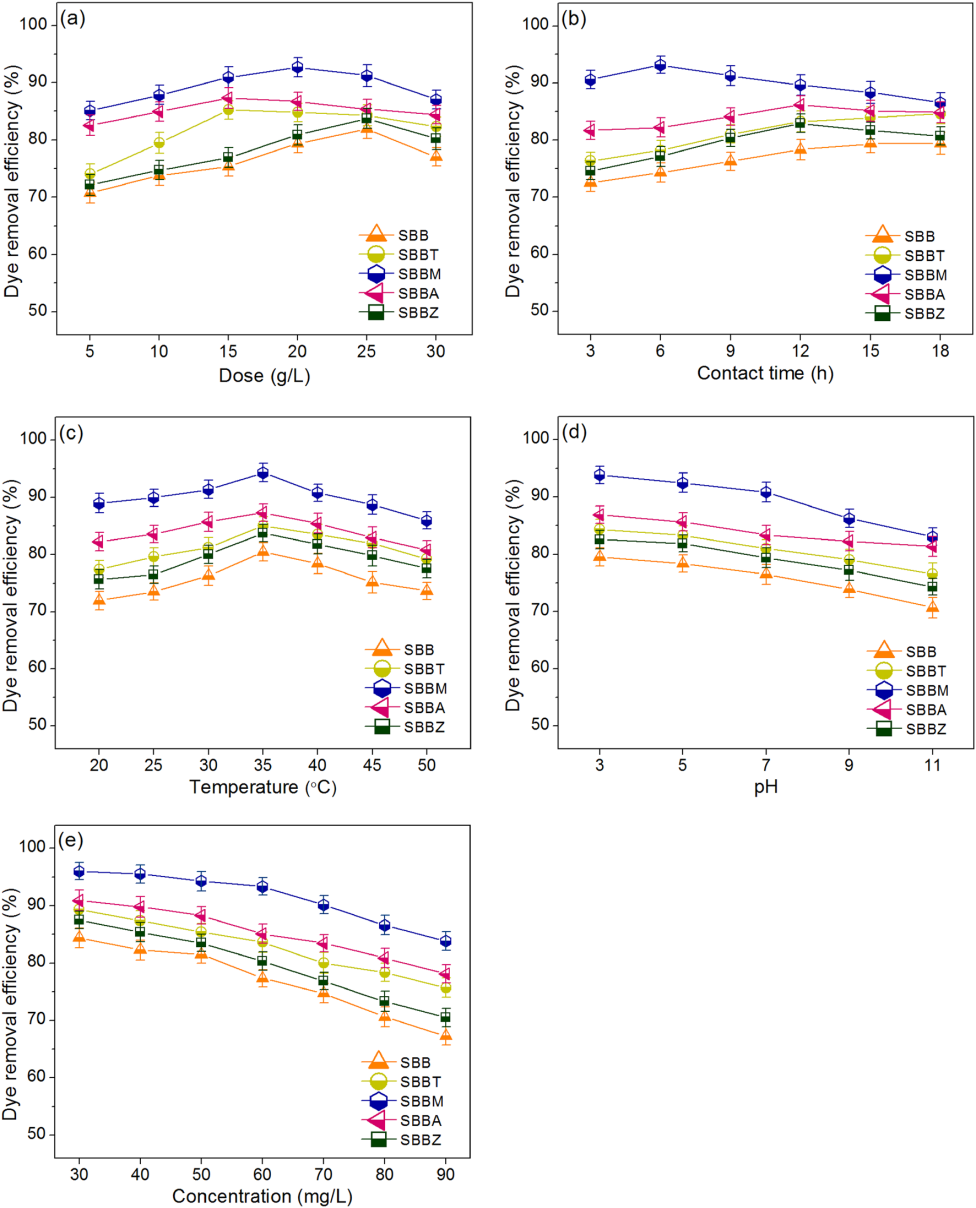
The batch experiments of SBB, SBBT, SBBM, SBBA, and SBBZ in (**a**) dose, (**b**) contact time, (**c**) temperature, (**d**) pH, and (**e**) concentration for DR28 dye adsorptions.

The effect of dosage from 5 to 30 g/L was designed to investigate how many grams of each material are needed for adsorbing DR28 dye at a concentration of 50 mg/L, a sample volume of 100 mL, a contact time of 12 h, a pH 7, a temperature of 30 °C, and a shaking speed of 150 rpm^9^ to obtain the highest DR28 dye removal efficiency, and the results are shown in Fig. 5a. DR28 dye removal efficiencies of SBB, SBBT, SBBM, SBBA, and SBBZ were increased with increasing material dosage from 5 to 30 g/L because of increasing of active sites for adsorbing DR28 dye similarly reported by other studies^41,42^. Furthermore, the highest DR28 dye removal efficiencies were found at 25 g/L (81.90%), 15 g/L (85.23%), 20 g/L (92.67%), 15 g/L (87.30%), and 25 g/L (83.73%) for SBB, SBBT, SBBM, SBBA, and SBBZ, respectively. Therefore, they were used as the optimum dosages for the effect of contact time.

#### The effect of contact time

The effect of contact time from 3 to 18 h was used to determine how much contact time of each material is enough for adsorbing DR28 dye at a concentration of 50 mg/L, a sample volume of 100 mL, a pH 7, a temperature of 30 °C, a shaking speed of 150 rpm^9^, and the optimum contact dosage to achieve the highest DR28 dye removal efficiency, and the results are shown in Fig. 5b. DR28 dye removal efficiencies of SBB, SBBT, SBBM, SBBA, and SBBZ were increased with increasing contact time from 3 to 18 h until their saturated adsorptions with discovering constant contact time were the optimum contact time^18^. The highest DR28 dye removal efficiencies were found at 18 h (79.41%), 18 h (84.59%), 6 h (93.16%), 12 h (86.71%), and 12 h (82.94%) for SBB, SBBT, SBBM, SBBA, and SBBZ, respectively. Therefore, they were used as the optimum contact time for the effect of temperature.

#### The effect of temperature

The effect of temperature from 20 to 50 °C was examined how many temperatures of each material are good for adsorbing DR28 dye at a concentration of 50 mg/L, a sample volume of 100 mL, a pH 7, a shaking speed of 150 rpm^9^, and the optimum dosage and contact time to get the highest DR28 dye removal efficiency, and the results are shown in Fig. 5c. DR28 dye removal efficiencies of SBB, SBBT, SBBM, SBBA, and SBBZ were increased with the increases of temperature from 20 to 35 °C, and then they a little decreased. The highest DR28 dye removal efficiencies were found at 35 °C in all materials with 80.43%, 85.02%, 94.33%, 87.33%, and 83.75% for SBB, SBBT, SBBM, SBBA, and SBBZ, respectively. Therefore, a temperature of 35 °C was the optimum temperature for the effect of pH.

#### The effect of pH

The effect of pH from 3 to 11 was used to examine the influence of pH on DR28 dye removal efficiencies of SBB, SBBT, SBBM, SBBA, and SBBZ to find the optimum pH for adsorb DR28 dye at a concentration of 50 mg/L, a sample volume of 100 mL, a shaking speed of 150 rpm^9^, and the optimum dosage, contact time, and temperature to get the highest DR28 dye removal efficiency, and the results are shown in Fig. 5d. For pK_a_ and pH of solution (pH_solution_), if the pH_solution_ is higher than pK_a_ (pH_solution_ > pK_a_), the dye molecule is in an anionic form. On the opposite, if the pH_solution_ is less than pK_a_ (pH_solution_ < pK_a_), the dye molecule is in a cationic form. Since the pK_a_ of DR28 dye is 4.1^43^, the DR28 dye molecule should adsorb at pH_solution_ > pK_a._ From the results of the point of zero charges (pH_pzc_), their DR28 dye adsorptions should occur at pH_solution_ < pH_pzc_. As a result, the high DR28 dye adsorption of each material should be observed at pK_a_ < pH_solution_ < pH_pzc_. In Fig. 5d, their DR28 dye adsorptions were highly adsorbed at pH 3–5, and the highest DR28 dye removal efficiency was found at pH 3 with 79.56%, 84.35%, 93.83%, 86.87%, and 82.58% for SBB, SBBT, SBBM, SBBA, and SBBZ, respectively which might support by the pK_a_ of carboxyl group (–COOH) in materials which is 3–5^44^. In addition, these results also agreed with the prior studies that found the highest anionic dye removal efficiencies at pH 3^8,9,18,40^. Therefore, pH 3 was the optimum pH for the effect of concentration.

#### The effect of concentration

The effect of concentration from 30 to 90 mg/L observed how many concentrations of each material could adsorb DR28 dye at a sample volume of 100 mL a shaking speed of 150 rpm^9^, and the optimum dosage, contact time, temperature, and pH to get the highest DR28 dye removal efficiency, and the results are shown in Fig. 5e. DR28 dye removal efficiencies of SBB, SBBT, SBBM, SBBA, and SBBZ were decreased with increasing concentration because the decrease of active sites for adsorbing DR28 dye similar to other studies^18^. Their DR28 dye removal efficiencies from 30 to 90 mg/L were 67.27–84.39%, 75.73–89.39%, 83.84–96.02%, 78.09–90.94%, and 70.47–87.50% for SBB, SBBT, SBBM, SBBA, and SBBZ, respectively, and their DR28 dye removal efficiencies at 50 mg/L were 81.51%, 85.44%, 94.27%, 88.31%, and 83.51% for SBB, SBBT, SBBM, SBBA, and SBBZ, respectively.

Finally, the optimum conditions in dosage, contact time, temperature, pH, and concentration of SBB, SBBT, SBBM, SBBA, and SBBZ were 25 g/L, 18 h, 35 °C, pH 3, 50 mg/L, 15 g/L, 18 h, 35 °C, pH 3, 50 mg/L, 20 g/L, 6 h, 35 °C, pH 3, 50 mg/L, 15 g/L, 12 h, 35 °C, pH 3, 50 mg/L, and 25 g/L, 12 h, 35 °C, pH 3, 50 mg/L, respectively. DR28 dye removal efficiencies could be arranged in order from high to low of SBBM > SBBA > SBBT > SBBZ > SBB, and SBBM had the highest DR28 dye removal efficiency with spending less material dosage and contact time than other materials similarly found by previous study with sugarcane bagasse fly ash beads modified with the same types of metal oxide with this study for DR28 dye adsorptions in aqueous solution^18^. Moreover, these results also corresponded to the results of BET analysis that SBBM had a higher surface area with smaller pore size than other materials, so it could adsorb DR 28 dye more than others. Therefore, the addition of metal oxides of magnesium oxide (MgO), titanium dioxide (TiO_2_), aluminum oxide (Al_2_O_3_), and zinc oxide (ZnO) increased material efficiencies for adsorbing DR28 dye, and SBBM was a high-potential material to further use for industrial wastewater treatment.

For the comparison with other anionic dye removals, the previous studies have used sugarcane bagasse or sugarcane bagasse fly ash beads with or without metal modifications of iron (III) oxide-hydroxide, ZnO, TiO_2_, MgO, and Al_2_O_3_ for removing reactive blue 4 (RB4) and DR28 dyes^8,9,18^, and the results demonstrated sugarcane bagasse and sugarcane bagasse fly ash beads mixed MgO had the highest RB4 and DR28 dye removals than other materials. These results corresponded to this study that SBBM illustrated the highest DR28 dye removal, so it could confirm that sugarcane bagasse beads with or without metal modifications especially MgO could remove various anionic dyes of RB4 and DR28.

### Adsorption isotherms

**Figure 6:**
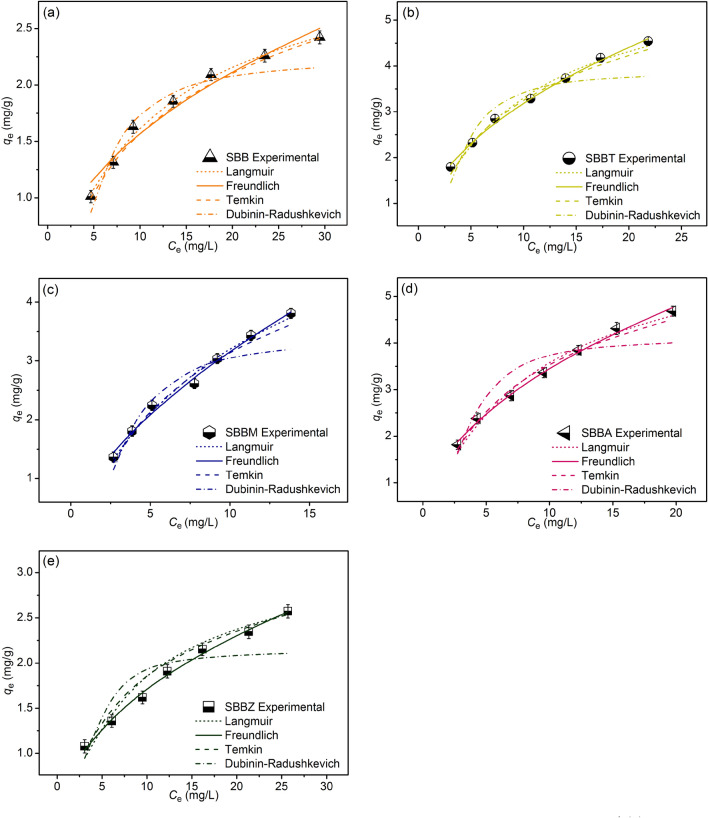
The adsorption isotherms of (**a**) SBB, (**b**) SBBT, (**c**) SBBM, (**d**) SBBA, and (**e**) SBBZ for DR28 dye adsorptions.

The adsorption patterns of SBB, SBBT, SBBM, SBBA, and SBBZ are described by various adsorption isotherms of Langmuir, Freundlich, Temkin, and Dubinin–Radushkevich models. Their graphs are plotted by *q*_e_ versus* C*_e_. The results are shown in Fig. 6a–e, and Table 4 displayed their equilibrium isotherm parameters.

**Table 4:** The equilibrium isotherm parameters of SBB, SBBT, SBBM, SBBA, and SBBZ for DR28 dye adsorptions.

Isotherm models	Parameters	SBB	SBBT	SBBM	SBBA	SBBZ
Langmuir	*q*_m_ (mg/g)	3.243	6.249	6.578	6.409	3.293
	*K*_L_ (L/mg)	0.101	0.119	0.095	0.128	0.131
	*R*^2^	0.997	0.989	0.983	0.988	0.983
	*R*^2^_adj_	0.996	0.987	0.980	0.986	0.979
	RMSE	0.038	0.118	0.101	0.142	0.142
Freundlich	1*/n*	0.428	0.464	0.594	0.471	0.427
	*K*_F_ (mg/g)(L/mg)^1/n^	0.588	1.100	0.804	1.168	0.643
	*R*^2^	0.971	0.998	0.992	0.997	0.994
	*R*^2^_adj_	0.965	0.997	0.991	0.996	0.993
	RMSE	0.096	0.058	0.086	0.071	0.045
Temkin	*b*_T_ (J/mol)	3234.628	1826.036	1761.978	1789.554	3635.924
	*A*_T_ (L/g)	0.823	1.139	0.919	1.246	1.239
	*R*^2^	0.991	0.990	0.988	0.988	0.972
	*R*^2^_adj_	0.990	0.988	0.986	0.985	0.966
	RMSE	0.069	0.133	0.144	0.165	0.152
Dubinin–Radushkevich	*q*_*m*_ (mg/g)	2.216	3.871	3.371	4.097	2.138
	*K*_DR_ (mol^2^/J^2^)	3.793	1.881	1.632	1.452	1.613
	*E* (kJ/mol)	0.363	0.516	0.554	0.587	0.557
	*R*^2^	0.921	0.841	0.906	0.860	0.746
	*R*^2^_adj_	0.905	0.809	0.887	0.832	0.695
	RMSE	0.168	0.480	0.352	0.490	0.323

The *R*^2^ value is normally used for determining which adsorption isotherm better explains the adsorption pattern, and the higher *R*^2^ is chosen. As a result, SBB corresponded to Langmuir model relating to the physical adsorption with a high *R*^2^ of 0.997, whereas SBBT, SBBM, SBBA, and SBBZ corresponded to Freundlich model relating to the chemisorption with heterogeneous adsorption with high *R*^2^ values of 0.998, 0.992, 0.997, and 0.994, respectively similar found in a previous study^18^.

Finally, the comparison of the maximum dye adsorption capacity (*q*_m_) of this study with other agriculture wastes for DR28 dye removals is demonstrated in Table 5. The *q*_*m*_ values of SBB, SBBT, SBBM, SBBA, and SBBZ were higher than cabbage (2.31 mg/g) and rice husk (1.28–2.04 mg/g)^15,45^, and the *q*_*m*_ value of SBBM had higher than prior studies in Table 5 expect the studies of Rehman et al.^46^, Ibrahim and Sani^47^, and Masoudian et al.^48^.

**Table 5:** The comparison of the maximum dye adsorption capacity (*q*_m_) with various agriculture wastes for DR28 dye removals.

Materials	*q*_m_ (mg/g)	References
Pine bark	3.92	^49^
Sugarcane bagasse treated with propionic acid	3.54	^50^
Cabbage	2.31	^45^
Potato peel	6.90	^46^
Pea peels	16.40	^46^
Watermelon rind	24.75	^47^
Watermelon rind modified by titanium oxide	15.30	^48^
Rice husk	1.58	^15^
Rice husk (biochar)	1.28	^15^
Rice husk (biochar) modified with potassium hydroxide (KOH)	2.04	^15^
SBB	3.24	This study
SBBT	6.25	This study
SBBM	6.58	This study
SBBA	6.41	This study
SBBZ	3.29	This study

### Adsorption kinetics

**Figure 7:**
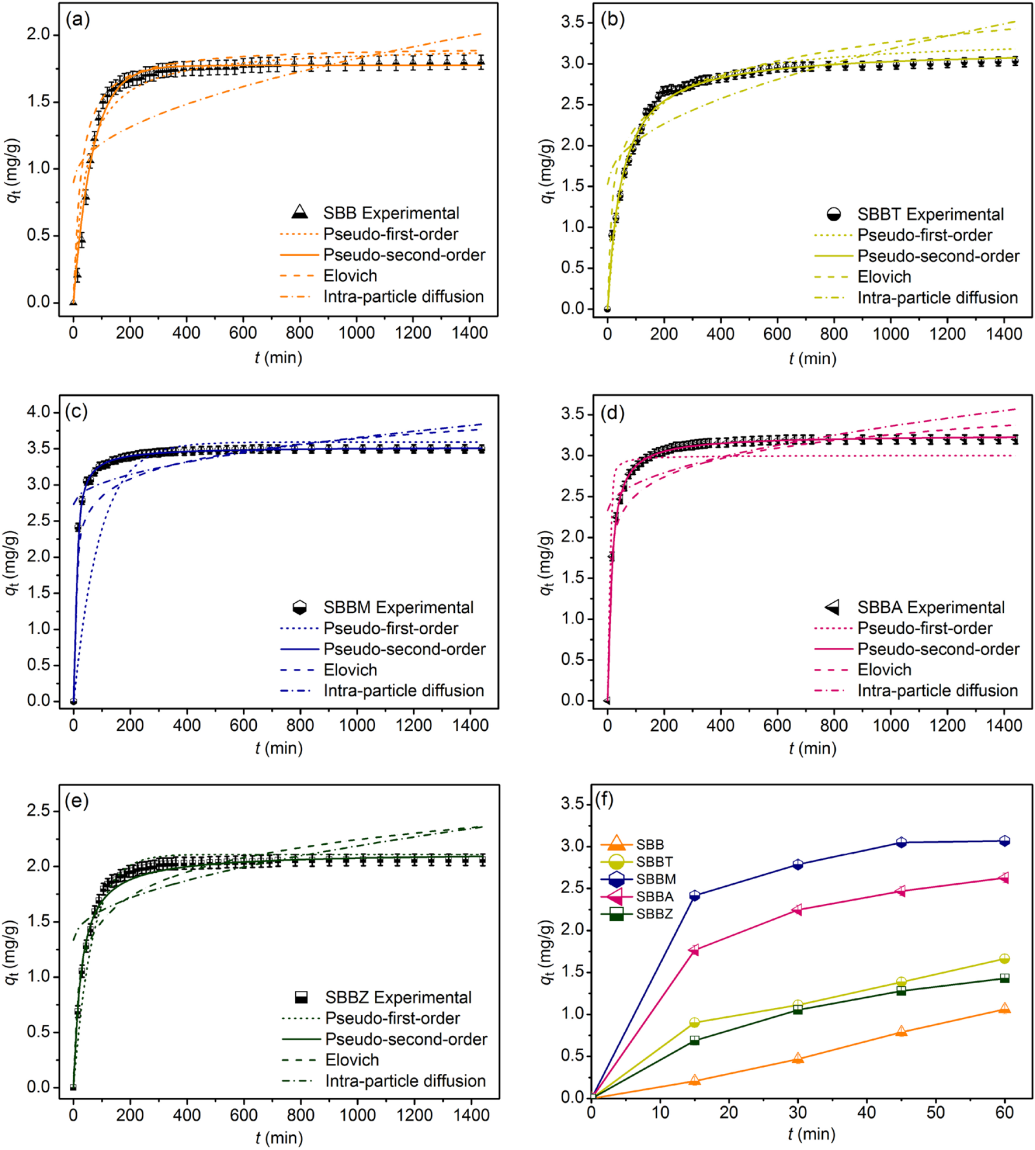
The adsorption kinetics of (**a**) SBB, (**b**) SBBT, (**c**) SBBM, (**d**) SBBA, (**e**) SBBZ for DR28 dye adsorptions, and (**f**) the equilibrium DR28 dye adsorption capacities of SBB, SBBT, SBBM, SBBA, and SBBZ.

The adsorption rates and mechanisms of SBB, SBBT, SBBM, SBBA, and SBBZ are determined by several adsorption kinetics of pseudo-first-order kinetic, pseudo-second-order kinetic, Elovich, and intra-particle diffusion models. Their graphs are plotted by *q*_t_ versus *t*. The results are shown in Fig. 7a–e, and Table 6 reported their equilibrium kinetic parameters.

**Table 6:** The adsorption kinetic parameters of SBB, SBBT, SBBM, SBBA, and SBBZ for DR28 dye adsorptions.

Kinetic models	Parameters	SBB	SBBT	SBBM	SBBA	SBBZ
Pseudo-first-order	*q*_e_ (mg/g)	0.438	1.091	1.819	1.252	0.447
	*k*_1_ (min^−1^)	0.049	0.002	0.003	0.003	0.007
	*R*^2^	0.917	0.702	0.731	0.721	0.941
	*R*^2^_adj_	0.915	0.696	0.725	0.715	0.940
	RMSE	1.244	0.511	0.577	0.274	0.095
Pseudo-second-order	*q*_e_ (mg/g)	1.922	3.178	3.526	3.254	2.151
	*k*_2_ (g/mg·min)	0.012	0.019	0.018	0.083	0.118
	*R*^2^	0.997	0.997	0.999	0.999	0.994
	*R*^2^_adj_	0.996	0.9960	0.999	0.999	0.993
	RMSE	0.094	0.060	0.018	0.017	0.049
Elovich	*α* (mg/g·min)	1.325	1.045	11.910	9.548	2.094
	*β* (g/mg)	3.864	2.225	4.346	3.240	3.961
	*R*^2^	0.714	0.922	0.769	0.814	0.837
	*R*^2^_adj_	0.708	0.921	0.764	0.810	0.833
	RMSE	22.506	0.225	0.254	0.224	0.180
Intra-particle diffusion	*k*_i_ (mg/g·min^0.5^)	0.030	0.058	0.029	0.033	0.013
	*C*_i_ (mg/g)	0.972	1.627	1.734	2.329	1.881
	*R*^2^	0.770	0.636	0.794	0.762	0.727
	*R*^2^_adj_	0.765	0.628	0.790	0.757	0.721
	RMSE	0.295	0.387	0.448	0.412	0.291

Similar to adsorption isotherm, the *R*^2^ value is normally used for determining which adsorption kinetic better describes the adsorption rate and mechanism, and the higher *R*^2^ is preferred. Since the *R*^2^ values of SBB, SBBT, SBBM, SBBA, and SBBZ in a pseudo-second-order kinetic model demonstrated the highest values of 0.997, 0.997, 0.999, 0.999, and 0.994, respectively, their adsorption rates and mechanisms were well described by chemisorption with the heterogeneous process agreed with a previous study reported^18^. In addition, the kinetic parameter of *q*_e_ is used for comparing their DR28 dye adsorption capacities. The *q*_e_ of SBBM was higher than other materials, so it could adsorb DR28 dye more than other materials agreed with the batch experiment results. Furthermore, the equilibrium DR28 dye adsorption capacities of SBB, SBBT, SBBM, SBBA, and SBBZ demonstrated in Fig. 7f which reached the equilibrium within 60 min indicated their fast kinetic reaction rates.

### Thermodynamic study

**Figure 8:**
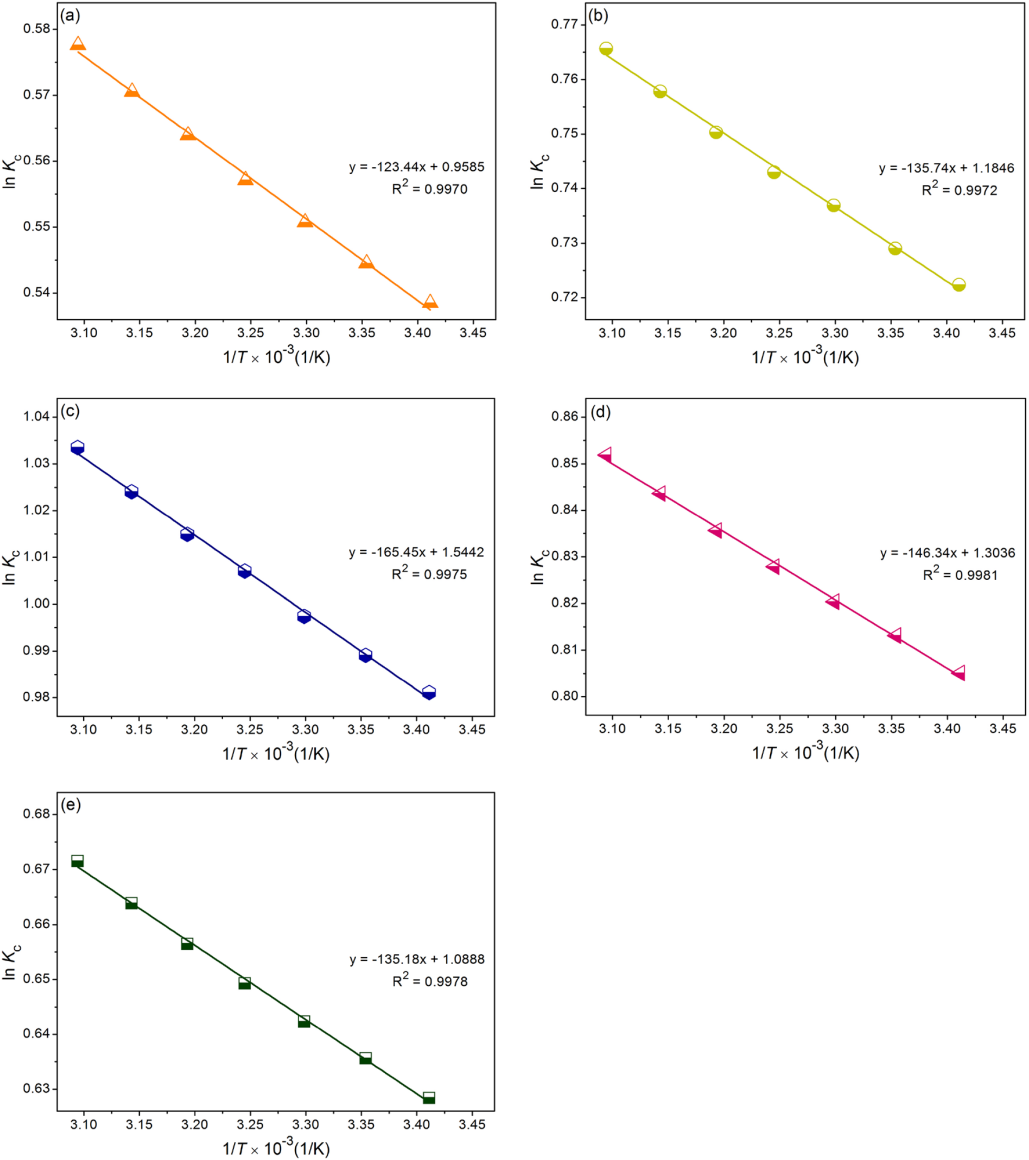
The thermodynamic plots for DR28 dye adsorptions by (**a**) SBB, (**b**) SBBT, (**c**) SBBM, (**d**) SBBA, and (**e**) SBBZ.

The results of thermodynamic studies in a range of 293.15–323.15 K of SBB, SBBT, SBBM, SBBA, and SBBZ on DR28 dye removals are demonstrated in Table 7 and Fig. 8a–e. Their ∆*G*° had negative values in all temperatures which meant they were a favorable adsorption process of a spontaneous nature. For ∆*H*°, all materials had positive values which meant their DR28 dye adsorption processes were endothermic^18^, and their ∆*S*° had positive values which meant the randomness during the adsorption process was increased^51^. Therefore, the increasing temperature was favorable for DR28 dye adsorptions onto all materials.

**Table 7:** Thermodynamic parameters of SBB, SBBT, SBBM, SBBA, and SBBZ**.**

	SBB	SBBT	SBBM	SBBA	SBBZ
Δ*G*° (J/mol)					
293.15 K	− 1312.45	− 1760.65	− 2391.08	− 1962.24	− 1531.55
298.15 K	− 1349.72	− 1807.15	− 2451.84	− 2015.61	− 1575.55
303.15 K	− 1387.98	− 1857.25	− 2513.91	− 2067.68	− 1618.91
308.15 K	− 1427.26	− 1903.46	− 2579.93	− 2140.78	− 1663.39
313.15 K	− 1468.15	− 1953.38	− 2642.27	− 2175.51	− 1709.05
318.15 K	− 1509.13	− 2004.55	− 2708.70	− 2231.39	− 1755.93
323.15 K	− 1551.83	− 2057.05	− 2776.74	− 2288.66	− 1804.11
Δ*H*° (J/mol)	1026.28	1128.54	1375.55	1216.67	1123.89
Δ*S*° (J/mol K)	7.97	9.85	12.84	10.84	9.05

## The possible mechanisms for DR28 dye adsorptions

**Figure 9:**
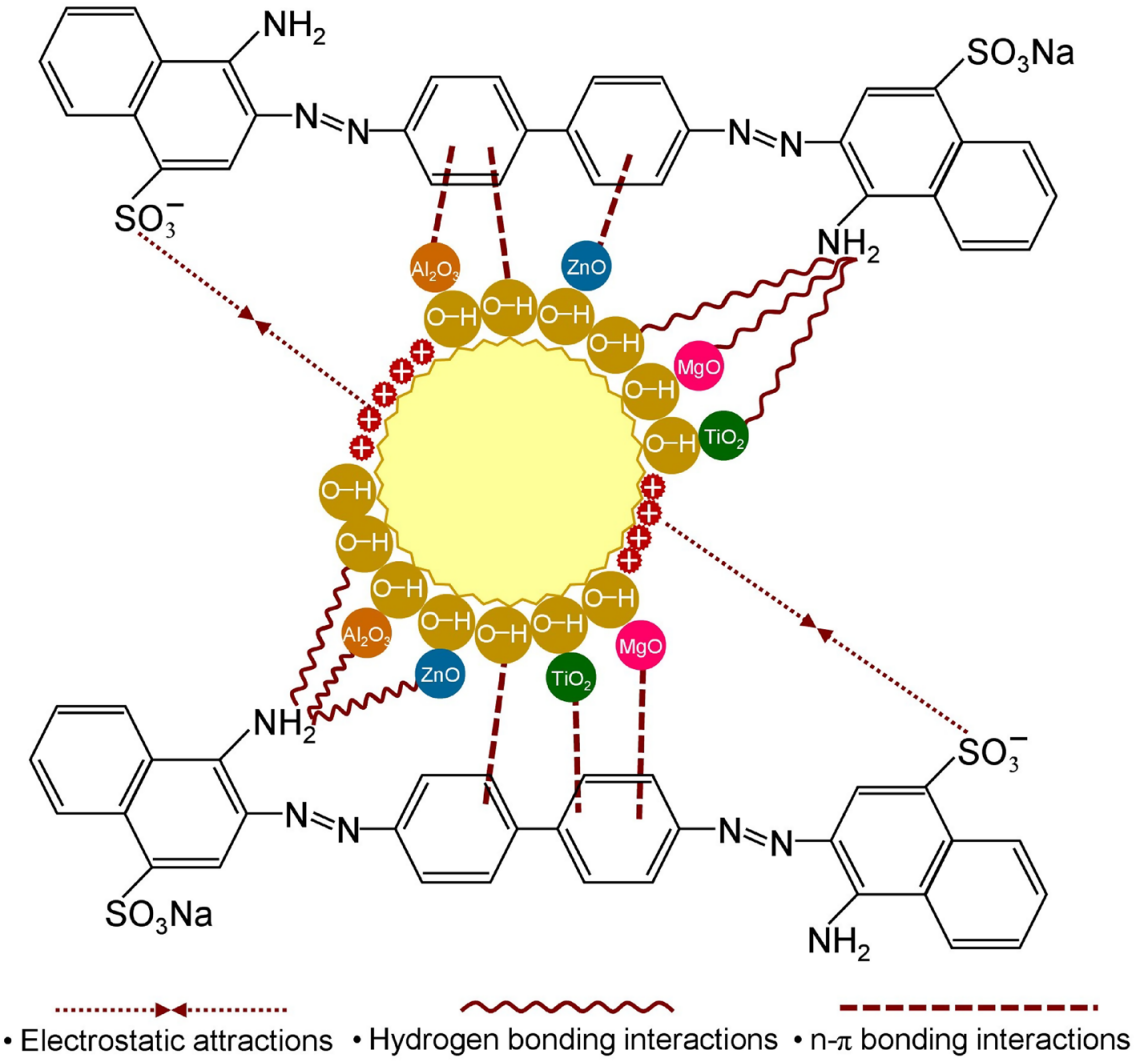
The possible mechanisms for DR28 dye adsorptions by SBB, SBBT, SBBM, SBBA, and SBBZ.

The possible mechanisms for DR28 dye adsorptions of SBB, SBBT, SBBM, SBBA, and SBBZ are demonstrated in Fig. 9 which modified the idea from the study of Ngamsurach et al.^8^ and Praipipat et al.^9,18^. Their main chemical functional groups of O–H, C–H, C=O, C=C, and C–O–C**,** and the complex molecules of Ti–O–Ti, Mg–O, Al–O–Al, and Zn–O connected with their hydroxyl group (O–H) played a main role for DR28 dye adsorptions. The possible mechanisms of electrostatic attraction, hydrogen bonding interaction, and n–π bonding interaction are used for explaining DR28 dye adsorptions by SBB, SBBT, SBBM, SBBA, and SBBZ demonstrated in Fig. 9.

## Conclusion

Five adsorbent materials of sugarcane bagasse beads (SBB), sugarcane bagasse modified with titanium dioxide beads (SBBT), sugarcane bagasse modified with magnesium oxide beads (SBBM), sugarcane bagasse modified with aluminum oxide beads (SBBA), and sugarcane bagasse modified with zinc oxide beads (SBBZ) were synthesized from sugarcane bagasse and various metal oxides for investigating their DR28 dye removal efficiencies. SBBM had the highest specific surface area and pore volume, whereas its pore size was the smallest among other materials. The surfaces of SBB, SBBM, SBBT, and SBBA were scaly sheet surfaces and structures with an irregular shape, whereas SBBZ was a coarse surface. Five main chemical elements of oxygen (O), carbon (C), calcium (Ca), chloride (Cl), and sodium (Na) were observed in all materials, whereas titanium (Ti), magnesium (Mg), aluminum (Al), and zinc (Zn) only detected in SBBT, SBBM, SBBA, and SBBZ, respectively. Five main chemical functional groups of O–H, C–H, C=O, C=C, and C–O–C were found in all materials, and Ti–O–Ti, Mg–O, Al–O, and Zn–O were observed in SBBT, SBBM, SBBA, and SBBZ. The points of zero charge (pH_pzc_) of SBB, SBBT, SBBM, SBBA, and SBBZ were 6.57, 7.31, 10.11, 7.25, and 7.77, respectively. All materials could adsorb DR28 dye at a concentration of 50 mg/L by more than 81%, and SBBM illustrated the highest DR28 dye removal efficiency of 94.27%. For adsorption isotherm, Langmuir model was a suitable model for SBB corresponding to physical adsorption, whereas Freundlich model was an appropriate model to explain the adsorption pattern of SBBT, SBBM, SBBA, and SBBZ relating to physicochemical adsorption. For adsorption kinetic, a pseudo-second-order kinetic model was the best-fit model for all materials well explained by the chemisorption mechanism. Since the ∆*G*° of all materials had negative values, they were a favorable adsorption process of a spontaneous nature. While their ∆*H*° had positive values which meant they were an endothermic process. For ∆*S*°, they had positive values which meant the randomness during the adsorption process was increased. Therefore, all materials were potential materials for adsorbing DR28 dye, especially SBBM.

For future works, the real wastewater might be applied to confirm their abilities for DR28 dye adsorptions. In addition, other anionic dyes might be investigated for possible adsorption by SBB, SBBT, SBBM, SBBA, and SBBZ. Moreover, the continuous flow study should study for the possible application in the industrial wastewater system. Furthermore, the leaching of metal oxides from SBBT, SBBM, SBBA, and SBBZ after the adsorption process might be suggested to investigate and confirm their no contaminations in treated wastewater.

## Data Availability

The datasets used and/or analyzed during the current study are available from the corresponding author upon reasonable request.
